# Changes in the Cellular Distribution of Tyrosine Phosphorylation and Its Relationship with the Acrosomal Exocytosis and Plasma Membrane Integrity during In Vitro Capacitation of Frozen/Thawed Bull Spermatozoa

**DOI:** 10.3390/ijms21082725

**Published:** 2020-04-15

**Authors:** Sara Ruiz-Díaz, Sergio Grande-Pérez, Sol Arce-López, Carolina Tamargo, Carlos Olegario Hidalgo, Serafín Pérez-Cerezales

**Affiliations:** 1 Department of Animal Reproduction, The National Institute for Agricultural and Food Research and Technology (INIA) and Agrifood research, Ctra. de La Coruña km 5.9., 28040 Madrid, Spain; sruizd@clinicatambre.com (S.R.-D.); sergio.gr3@gmail.com (S.G.-P.); solarcelopez@hotmail.com (S.A.-L.); 2 Mistral Fertility Clinics S.L., Clínica Tambre, Calle Tambre, 8, 28002 Madrid, Spain; 3 Department of Animal Selection and Reproduction, The Regional Agri-Food Research and Development Service of Asturias (SERIDA), Camino de Rioseco 1225, La Olla, Deva, E-33394 Gijón, Spain; ctamargo@serida.org (C.T.); cohidalgo@serida.org (C.O.H.)

**Keywords:** bull spermatozoa, protein tyrosine phosphorylation, membrane integrity, acrosome reaction, sperm capacitation

## Abstract

During sperm capacitation, intracellular signaling leads to protein tyrosine phosphorylation (PTP) of multiple cellular structures. However, the connection of this molecular signaling to the physiology of capacitated spermatozoa is not completely understood. This is the case of the short lifespan of capacitated spermatozoa and their increased susceptibility to initiate acrosomal exocytosis (AE) during incubation. Herein, by employing frozen/thawed bull spermatozoa, we aimed to study the relationship between PTP with AE and with plasma membrane integrity (PMI) at the cellular level. For this, we employed double staining following immunofluorescence for PTP combined with fluorescence probes for the acrosome (PNA-FITC) and PMI (LIVE/DEAD Fixable Dead Cell Stain Kit). Our results revealed that the presence of PTP at sperm head was less abundant in the sperm fraction that triggered the AE after 3 h of incubation under capacitating conditions, or by its induction with calcium ionophore, compared to the unreacted fraction. Furthermore, PTP at the equatorial region of the head (PTP-EQ) was enriched in the fraction showing damaged membrane while induction of AE with calcium ionophore did not alter the PMI and its relation to PTP-EQ. These results suggest that spontaneous AE and induced AE trigger similar cellular events regarding PTP and the spermatozoa showing PTP-EQ are more prone to suffer plasma membrane damage.

## 1. Introduction

Capacitation is a combination of physiological changes in the spermatozoa triggered within the female genital tract that is necessary for the acquisition of fertilizing competence [1]. This process comprises the activation of a complex intracellular signaling network [2,3] that leads to the phosphorylation of the tyrosine, serine, and threonine residues of multiple proteins at different cellular locations [1,4,5]. This signaling network is regulated by cAMP, protein kinase-A, as well as tyrosine kinase/phosphatase and serine/threonine kinase pathways [5,6]. Protein tyrosine phosphorylation (PTP) of the sperm flagellum plays an important role in regulating sperm motility to acquire hyperactivation, a hallmark of sperm capacitation [7,8]. Furthermore, PTP of the sperm head has been related to sperm–oocyte interaction and fusion events [8]. Different cellular patterns of PTP distribution in the spermatozoa have been described in many mammalian species, such as ram [9,10], mouse [11], human [12,13,14], boar [15], equine [16], buffalo [17,18], and also in bull, during sperm maturation in the epididymis [19] and in fresh and cryopreserved semen [18,20,21,22,23]. These studies aimed to analyze this PTP distribution together with other capacitation events in response to different treatments and conditions. However, the direct relation in each individual cell between PTP patterns and other capacitation events, such as plasma membrane integrity or acrosomal exocytosis, was not addressed.

During capacitation, the plasma membrane loses its cholesterol content and undergoes glycoprotein redistribution while phospholipid asymmetry is lost and phospholipase activity is increased [24]. As a consequence, capacitation reduces the longevity of the spermatozoa [25]. Thus, motility and viability decrease under capacitating conditions during the time [10]. One of the processes involved in this viability decrease is related to cell death via reactive oxygen species (ROS) signaling [26]. Intracellular ROS directly stimulates the adenylyl cyclase provoking downstream increase of PTP for subsequently reaching capacitation [27]. However, when this oxidative stress associated to capacitation exceeds a certain limit the cell undergoes apoptosis [26].

Another cellular component affected during sperm capacitation and highly related to the membrane integrity is the acrosome. This is an exocytotic vesicle located at the apical region of the mammalian sperm head containing hydrolytic enzymes that are released after the fusion of the acrosomal and plasma membrane [28], a process known as acrosomal exocytosis (AE). The release of these hydrolytic enzymes is thought to facilitate the sperm penetration of the cumulus oocyte complex [29]. However, recent publications have shown that mouse spermatozoa that reach the fertilization site have already undergone AE [30] and the same seems to occur in cattle [31,32,33]. The AE is a calcium-dependent exocytotic event [28] that can be artificially induced using calcium ionophores, such as ionomycin and A23187, and also with physiological molecules, such as progesterone or glycoproteins from the zona pellucida of some species [34,35]. Only after incubation under capacitating conditions, a fraction of the spermatozoa becomes susceptible to induced AE being proposed to be the fraction of capacitated spermatozoa [36]. It is important to point out that, by opening of the sperm calcium channels, the ionophores induce both capacitation and subsequently AE [37].

Herein, we aimed to study the relationship between the cellular distribution of PTP with the AE and plasma membrane integrity (PMI) in bull spermatozoa by employing fluorescence double staining approaches. We report here the loss of PTP in the head of those spermatozoa showing spontaneous AE after 3 h of incubation or after a treatment with the ionophore A23187. In addition, we show the existence of a direct relationship between the compromised plasma membrane and the presence of PTP in the equatorial region of the sperm head.

## 2. Results

### 2.1. Overall Effects of Processing and Incubation on Analyzed Semen Characteristics

To study the relationship between PTP with AE and with PMI we employed frozen/thawed sperm samples from six bulls that were washed by density gradient centrifugation (DGC) and incubated for three hours under capacitating conditions. Computer assisted sperm analysis (CASA) [38] revealed no significant differences in the sperm motility or kinetics between the sample before DGC and the sample at time 0 of incubation (Table 1). However, after two hours of incubation, we detected a significant decrease in the percentage of motile spermatozoa accompanied by an increase in the linearity of the trajectories, as reflected by a raise of the parameters LIN and STR (Table 1). These kinetics were maintained to the same levels until the three hours of incubation (Table 1). Besides, we recorded an increase of the beat cross frequency (BCF) after 3 h of incubation (Table 1). Analysis of the AE and PMI, employing the fluorescence probes PNA-FITC [39] and LIVE/DEAD Fixable Dead Cell Stain Kit (Invitrogen Life Technologies, Carlsbad, CA, USA) respectively, also revealed no significant differences between the sample before DGC and the control at time 0 (Table 2). However, after three hours of incubation, we detected a significant rise in spontaneous AE and a decrease in PMI (Table 2).

**Figure 1:**
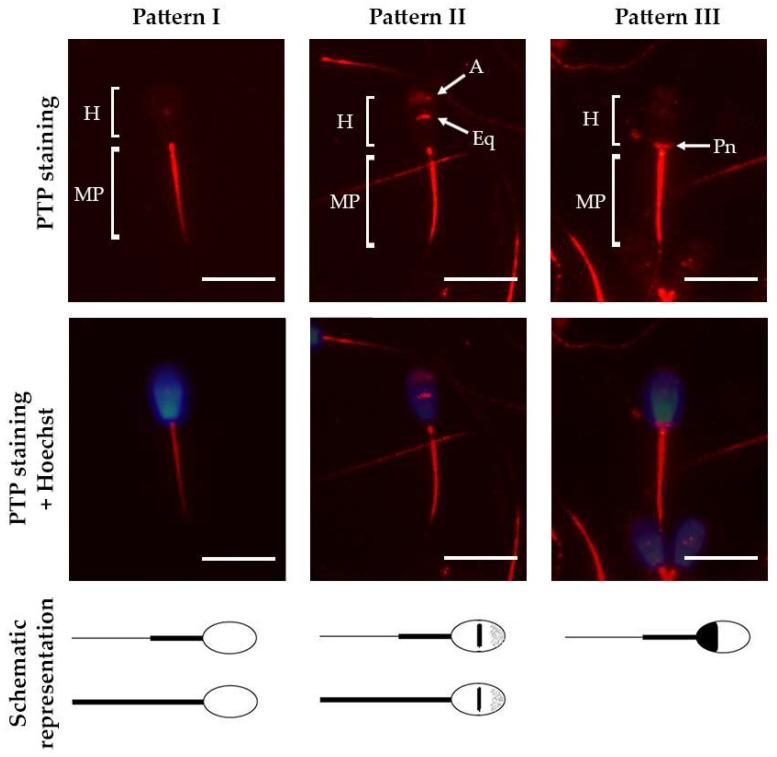
Representative micrographs of the three PTP patterns in bull spermatozoa that were detected employing immunolabeling (red). Nuclei were counterstained with Hoechst 33342 (blue). Below the micrographs, a schematic representation of each pattern is shown. Pattern I: staining at the midpiece (MP) and/or the whole flagella; pattern II: staining at the acrosomal region (A), the equatorial region (Eq), the midpiece and/or the whole flagella; pattern III: staining at the post-nuclear region (Pn) and the midpiece. Scale bar represents 10 µm.

Immunofluorescence analysis revealed PTP at various sperm locations (Figure 1). While every analyzed spermatozoon showed PTP at the midpiece, we observed three clear staining patters involving the equatorial and the post-nuclear regions of the head (Figure 1). Less than 10% of the spermatozoa also showed PTP of the whole flagellum (Table 2).

Considering the percentage of spermatozoa showing PTP at any location of the head (pattern II + III), or only in the equatorial region (pattern II), or in the whole flagellum, we did not find significant differences in staining between the sample before DGC and the control after DGC at time 0 (Table 2). However, we registered a significant increase of PTP after three hours of incubation (*p* < 0.05), in the equatorial region and the whole flagella (Table 2). Together with changes in kinetics, these results suggest the occurrence of sperm capacitation within the duration of the experiment. 

To confirm the capacitated status of the spermatozoa we employed the calcium ionophore A23187 to induce the AE as described elsewhere [36]. This experiment showed that, at the beginning of the incubation (time 0), the AE was not induced by the ionophore, but after three hours of incubation, the ionophore caused a rise in the percentage of acrosome reacted spermatozoa with respect to the control (Table 2). These results also confirm the capacitated status of the spermatozoa after three hours of incubation, revealing at this time point a fraction of spermatozoa susceptible to induced AE [36]. However, this fraction was small (3.3 ± 2.5%) when compared to other studies employing similar conditions [40] that could be explained by inter-individual and/or inter-breed variability. As a matter of fact, in the six bulls that we employed here, the response to the ionophore treatment after 3 h of incubation ranged from 1.3% to 7.7% of positive AE, demonstrating the existence of high inter-individual variability in the response. Longer incubation periods could increase the observed response as ionophore itself provokes capacitation and also triggers the subsequent AE [40].

Furthermore, we observed that the treatment with the ionophore did not disturb the membrane integrity or the PTP staining (Table 2). 

### 2.2. Relationship between Acrosomal Exocytosis and Cellular Distribution of Protein Tyrosine Phosphorylation

**Figure 2:**
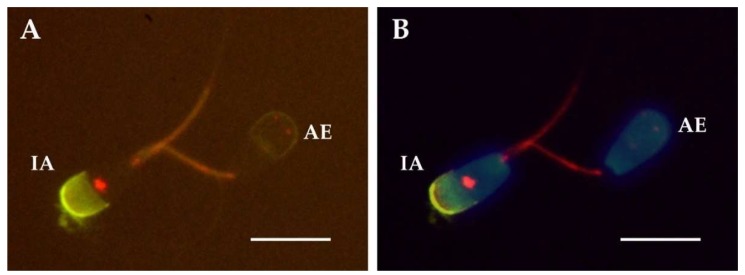
Representative micrographs of the staining employing immunolabeling for the detection of PTP (red-orange) and PNA-FITC for the acrosome (green). (**A**) micrograph taken with a fluorescence filter for visualizing PNA-FITC (green) together with the staining of PTP (red-orange). (**B**) micrograph merged after taking pictures with three filters: for the visualization of the nuclei stained with Hoechst 33342 (blue), PNA-FITC (green) and the staining of PTP (red). Spermatozoon showing intact acrosome (IA) and the PTP staining pattern II and spermatozoa showing acrosomal exocytosis (AE) and the PTP staining pattern I. Scale bar represents 10 µm.

**Figure 3:**
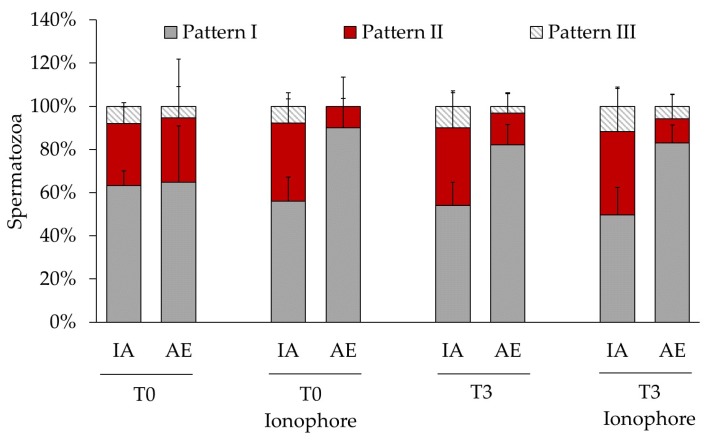
Distribution of protein tyrosine phosphorylation in bull spermatozoa showing intact acrosome (IA) or acrosomal exocytosis (AE). Spermatozoa were analyzed after a treatment with or without ionophore A23187 at time 0 (T0) or after 3 h (T3) of incubation under capacitating conditions. Values of each pattern are represented as means (%) ± SD, *n* = 6.

We employed double fluorescence staining for the detection of PTP and AE (Figure 2). We found significant differences in the abundance of each pattern between the sperm fractions showing AE and the fraction showing intact acrosome (Table 3, Figure 3). Thus, the pattern I, spermatozoa showing PTP only at the midpiece, was the most abundant in both fractions for most of the samples. Interestingly, after three hours of incubation this pattern was more abundant in the spermatozoa that suffered spontaneous AE. The same result was obtained when the sample was treated with calcium ionophore at both times 0 and 3 h. In contrast, the percentage of spermatozoa showing the staining patterns II (midpiece and equatorial region) and III (midpiece and postnuclear region) was significantly lower than the percentage of spermatozoa showing AE in these same samples.

### 2.3. Relationship between Membrane Integrity and Cellular Distribution of Protein Tyrosine Phosphorylation

**Figure 4:**
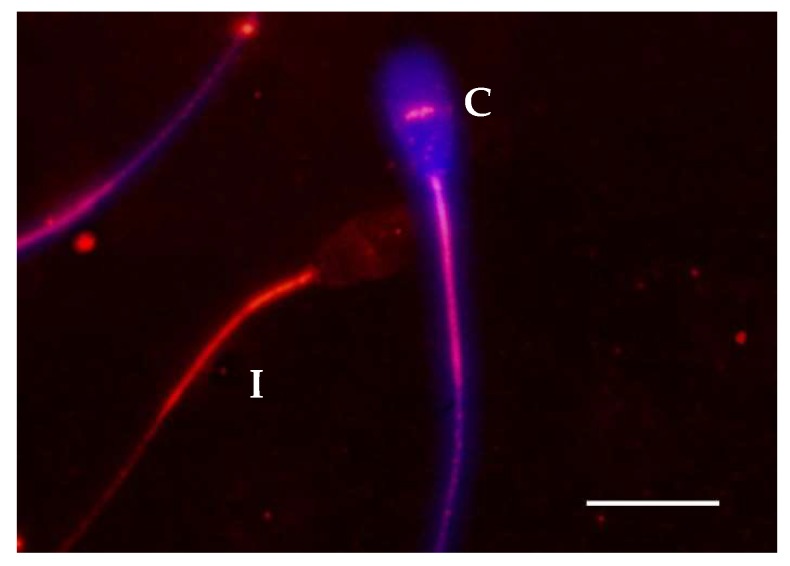
Representative micrograph of double staining employing immunolabeling for the detection of PTP (red) and membrane integrity staining (purple). Spermatozoa showing compromised sperm membrane (C) and PTP staining pattern II, and spermatozoon showing intact sperm membrane (I) and PTP staining pattern I. Scale bar represents 10 µm.

**Figure 5:**
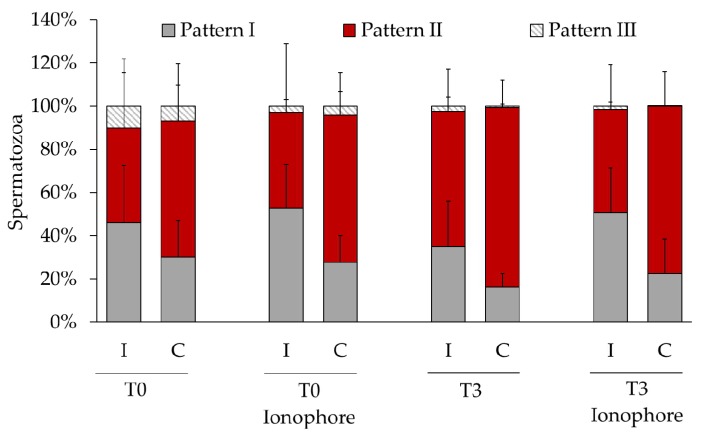
Distribution of protein tyrosine phosphorylation in bull spermatozoa showing spermatozoa with intact (I) or compromised (C) plasma membrane. Spermatozoa were analyzed after a treatment with or without ionophore A23187 at time 0 (T0) or after 3 h (T3) of incubation under capacitating conditions. Values of each pattern are represented as means (%) ± SD, *n* = 6.

Double staining of the PTP and PMI (Figure 4) revealed that, before the DGC, the patterns I and II were equally abundant in spermatozoa showing compromised and intact plasma membranes (Table 4). However, at the beginning and after three hours of incubation, the pattern II was significantly more abundant in spermatozoa with compromised plasma membrane and the pattern I was significantly more abundant in spermatozoa with intact plasma membrane (Table 4, Figure 5). These results indicate a physiological connection between the PMI and the occurrence of PTP in the equatorial region of the sperm head. We did not find any difference between the remaining staining patterns, and the ionophore did not affect the observed effect.

## 3. Discussion

Herein, we aimed to reveal the link between the dynamics of PTP during capacitation with the AE and the PMI. In our experiment, the incubation for 3 h under capacitating conditions induced the occurrence of capacitation-related events known to occur in bull spermatozoa such as an increase of the BCF [41] (Table 1), and enrichment of the PTP of the sperm head as well as of the flagellum [42] (Table 2). Furthermore, we confirmed successful capacitation by and increased susceptibility to induced AE by calcium ionophore after 3 h of incubation when compared to non-capacitated control (sample at the beginning of the incubation) [36] (Table 2).

In addition, total motility and PMI during the incubation period significantly decreased to similar values as previously reported in Canchim bull within the same incubation time [43]. Intracellular ROS generation associated with sperm capacitation has been pointed to as a major agent of this decrease of mammalian sperm PMI under capacitating conditions in vitro [26,27]. We have also detected an increase of spontaneous AE after 3 h of incubation as has been reported in other species when incubating with capacitation promoting media [44].

In this study, we have employed frozen/thawed spermatozoa. Cryopreservation increases plasma membrane permeability, leading to a calcium intake that raises the intracellular concentration of cAMP increasing PTP, and generating a capacitation-like state known as cryo-capacitation [8]. We did not see a relationship between spontaneous AE or PMI and PTP patterns in the spermatozoa right after thawing. Thus, spermatozoa showing AE or with compromised sperm plasma membrane show the same abundance of every PTP pattern than in spermatozoa with intact acrosome and plasma membrane (Table 3 and Table 4). These results cannot prove or rule out the occurrence of cryo-capacitation, since we did not have the possibility of analyzing the sperm status before freezing. However, the capacitation-related events registered during the three hours of incubation could have been facilitated by the process of freezing/thawing [45]. Thus, it is important to point out that spermatozoa from fresh ejaculates could behave differently under the same conditions.

After thawing, we registered around 40% of spermatozoa showing PTP on the sperm head (Table 2), and most of them at the equatorial region and acrosome (pattern II, Figure 1). This pattern has been previously described as the most abundant in spermatozoa retrieved from the cauda epididymis of Slovak spotted bulls [19] and the most abundant in the Japanese black bull’s ejaculate [23], showing wide differences in its abundance among individuals. Herein, we report a time-dependent increase of this pattern abundancy, demonstrating the acquisition of PTP in the head by those spermatozoa that were initially non-phosphorylated (Table 2). Furthermore, at the beginning of the incubation, we found that the spermatozoa lacking the acrosome showed the same PTP distribution than those with intact acrosome, while after three hours ~80% of the former ones lost the PTP in the head (Table 3, Figure 3). Our basal level of AE detected after thawing and at time 0 could be a consequence of the freezing/thawing process as it is known to cause acrosome loss [46]. Nevertheless, it is known that ejaculated semen has also a fraction of spermatozoa that have undergone AE, which could be another explanation for this initial levels of AE [47]. Interestingly, our results suggest that the acrosomal lost triggered by this process does not follow the same events than the spontaneous and ionophore-induced AE because, in contrast to these, PTP was not lost. Thus, the loss of PTP in the sperm head of spermatozoa that underwent spontaneous AE during capacitation could reflect a physiological phenomenon. The presence of well localized, non-diffused PTP in the sperm head, has been identified to function in stabilizing the acrosome integrity in bull [23] and therefore we hypothesize that the loss of this PTP could be a trigger of the AE. Perhaps overall protein dephosphorylation is a sine qua non condition for AE since dephosphorylation of the serine/threonine residues of proteins located in the post-acrosomal region is necessary for the occurrence of the acrosomal exocytosis in bull [48]. Another possibility is that the loss of PTP in the head after three hours of incubation is produced by the diffusion of the phosphorylated proteins from the acrosomal matrix as they might be cytosolic proteins [21].

We did not detect an overall increase of PTP produced by the treatment with the ionophore as reported in other species like humans [49]. In contrast, we found that calcium ionophore induced the loss of PTP in the head in those spermatozoa lacking the acrosome at the beginning of the incubation, while after three hours it did not produce an extra loss of PTP already produced by the spontaneous AE (Table 3, Figure 3). Why ionophore caused this loss of PTP is an intriguing question. As commented above, at the beginning of incubation the presence of a sperm population lacking the acrosome could have been produced by the freezing/thawing procedure, leaving intact the PTP that, according to our hypothesis, must be removed for the actual AE. Then, it is possible that the ionophore in these spermatozoa triggered some intracellular cascade, yet to identify, that leads to protein-tyrosine dephosphorylation for inducing the AE, as we think it was produced spontaneously during capacitation (Table 3, Figure 3).

Regarding the analysis of the PMI, we found that the spermatozoa with compromised plasma membrane were enriched in PTP at the equatorial region in every sample just right after thawing (before DGC) (Table 4, Figure 5). To our knowledge, this relationship has never been reported before and we hypothesize that it could indicate that these spermatozoa are at a late stage of capacitation as membrane becomes more fluid and permeable to vital stains such as shown for propidium iodide [50]. Another possibility is that capacitated spermatozoa, thus showing increased PTP, are more prone to enter in an apoptotic state and suffer membrane damage [26,27]. Perhaps both hypotheses are correct and connected, but further analysis would be needed to verify this possibility.

We conclude that frozen/thawed bull spermatozoa incubated in vitro for 3 h under capacitating conditions show the occurrence of capacitated related events. At the cellular level, we confirm a relationship between the loss of PTP in the equatorial region of the sperm head and both the occurrence of spontaneous and ionophore induced acrosome exocytosis. Furthermore, we demonstrated that the presence of PTP in the equatorial region of the sperm head is directly related to a compromised integrity of the sperm plasma membrane. The approach used here may be a valuable strategy to study the dynamics, sequence, and synchrony of the capacitation events at the level of individual cells.

## 4. Materials and Methods

### 4.1. Reagents

All reagents were purchased from Sigma–Aldrich (St. Louis, MO, USA) except where otherwise stated.

### 4.2. Sperm Processing

Frozen seminal samples from six different Asturian Valley bulls were used. Bulls were housed at the Cenero AI Centre [Regional Service of Agrifood Research and Development (SERIDA), Gijón, Spain], complying with all European Union regulations for animal husbandry. Additional approval from an ethical committee to conduct this study was not required. Animals were selected on the basis of being good breeders by having AI outcomes using frozen samples above 50% of non-return rates (62 ± 9%, *n* = 6). Semen was collected by artificial vagina (initial temperature of the water: 45 °C) After collection, the ejaculate was diluted with Bioxcell^®^ (IMV technologies, L’Aigle, France) at room temperature (22 °C) at a final sperm concentration of about 92 × 10^6^ spz/mL. The extended semen was cooled from 22 °C to 5 °C over a period of 1.5 h (at a cooling rate of approximately 0.2 °C/min) and then left at 5 °C for another additional 2.5 h. Then, it was packaged in 0.25 mL straws (23 × 10^6^ spz/straw), and frozen in liquid nitrogen vapors in a programmable freezer following the IMV Digit-cool standard curve for bovine semen (5 °C/min from +4 °C to −10 °C; 40 °C/min from −10 °C to −100 °C and 20 °C/min from −100 °C to −140 °C).

Two straws (0.25 mL) of each bull (same ejaculate) were thawed at 37 °C in a water bath for 40 s. Thawed semen was washed by density gradient centrifugation by placing it on top of a BoviPure gradient (Nidacon Laboratories AB, Göthenborg, Sweden) of 1 mL at 80% (lower layer) and 1 mL at 40% (upper layer) and centrifuging for 5 min at 290× *g*. The pellet was then resuspended in FERT (Tyrode’s medium with 25 mM bicarbonate, 22 mM sodium lactate, 1 mM sodium pyruvate, and 6 mg/mL fatty acid-free bovine serum albumin (BSA) supplemented with 10 mg/mL heparin sodium salt (Calbiochem, San Diego, CA, USA)) to a final concentration of 20 × 10^6^ spz/mL and incubated for three hours at 37 °C and 5% CO_2_ for capacitation.

### 4.3. Experimental Design

This study aimed to evaluate, in bull spermatozoa, the relationship between acrosomal exocytosis and plasma membrane integrity with the cellular distribution of protein tyrosine phosphorylation before and after 3 h of in vitro incubation under capacitating conditions and after inducing the acrosome exocytosis. Sperm motility and kinetics were analyzed right after thawing, after gradient centrifugation (T0h) and every hour until 3 h of incubation (T1h, T2h, T3h). Fluorescence probes were used for analysis by double staining the cellular location of protein tyrosine phosphorylation and acrosome exocytosis or the cellular location of protein tyrosine phosphorylation and the plasma membrane integrity. These analyses were conducted with spermatozoa treated at T0h and T3h with or without ionophore A23187 (10 µM final concentration) for 30 min at 37 °C and 5% CO_2_.

### 4.4. Motility Analysis

Sperm motility was evaluated placing 6 µL of semen on a Makler chamber on the stage heated to 37 °C of a Nikon Eclipse E400 (Nikon, Tokyo, Japan) fitted with a digital camera Basler acA1300–200uc (Basler, Ahrensburg, Germany). Videos of 1 sec were recorded at 60 frames per second using the software Pylon Viewer provided by Basler. At least three random fields per sample were recorded. Motility and sperm kinetics were analyzed using the free software ImageJ 1.x [51] with the plugin CASA_bmg following the instructions of the developers [52]. The parameters analyzed were as described by Mortimer et al. (2000) [38]: straight-line velocity (VSL; time-averaged velocity of the sperm head along a straight line from its first position to its last position, expressed in µm/s); curvilinear velocity (VCL; time-averaged velocity of the sperm head along its actual curvilinear path, expressed in µm/s); average path velocity (VAP; velocity over an average path generated by a roaming average between frames, expressed in µm/s); linearity (LIN) (defined as (VSL/VCL) × 100); straightness (STR) (defined as (VSL/VAP) × 100); wobble (WOB) (defined as (VAP/VCL) × 100); amplitude of lateral head displacement (ALH; width of the lateral movement of the sperm head, expressed in µm) and beat-cross frequency (BCF; number of times the sperm head crosses the direction of movement per second, expressed in Hz). For motility analysis, right after thawing, the sample was diluted in FERT to a final concentration of 20 × 10^6^ spz/mL.

### 4.5. Plasma Membrane Integrity Analysis

For PMI analysis, LIVE/DEAD Fixable Dead Cell Stain Kit (Invitrogen Life Technologies, Carlsbad, CA, USA) was used. The PMI dye was added to a final dilution of 1:1000 to 1 mL of PBS containing 1 × 10^6^ spermatozoa. Then, the samples were incubated for 30 min at 4 °C and centrifuged at 600× *g* for 5 min. The resultant pellet was resuspended in PBS and paraformaldehyde was added to a final concentration of 3.2% and incubated for 15 min at room temperature. After this, samples were washed twice at 600× *g* for 5 min in PBS discarding the supernatant. The last pellet was resuspended in 100 µL of PBS and stored at 4 °C until its processing for tyrosine phosphorylation staining when 50 µL of the sample was placed on a microscope slide and left to dry on a heater plate at 37 °C. Then immunodetection of tyrosine phosphorylation was conducted as described below. At least 200 spermatozoa per sample were analyzed.

### 4.6. Immunofluorescence Analysis of Protein Tyrosine Phosphorylation

Samples for double staining of PTP and AE were diluted to a total of 2 × 10^6^ spermatozoa in 500 µL of PBS. Then, the samples were centrifuged at 600× *g* for 5 min. The supernatant was discarded leaving 50 µL on the pellet that were subsequently mixed with 50 µL of 4% paraformaldehyde in PBS. The sample was left at room temperature for 10 min and then stored at −20 °C for its further analysis. After defrosting at room temperature, 200 µL of PBS was added and the sample was centrifuged at 1200× *g* for 5 min. This step was repeated twice more discarding the supernatant. After the last centrifugation, 30 µL of the pellet was left and smeared on a microscope slide and left to dry on a heater plate at 37 °C. 

For both double-staining experiments (PTP and AE or PTP and PMI) 200 µL of 0.2% triton X-100 in PBS were added on each dried slide placing a cover slide of 20 × 60 mm on top. The slides were then incubated for 15 min at 37 °C in a humid box after washing once with PBS, then 200 µL of 1% BSA in PBS were added and slides were incubated at 37 °C in a humid box for 1 h. After this, the slides were drained and 100 µL of the primary antibody (anti-Hu/Mo phosphotyrosine, clone pY20, Thermo Fischer Scientific, Walham, MA, USA) diluted at 1:100 were added covering with a cover slide and incubated at 4 °C overnight in a humid box. The next day, the slides were washed three times in PBS, and 100 µL of the secondary antibody (Alexa Fluor ^TM^ 594 goat anti-mouse IgG or Alexa Fluor^TM^ 488 Goat anti-mouse IgG for double staining for AE or PMI respectively, Invitrogen Life Technologies, Carlsbad, CA, USA) diluted at 1:100 in PBS were added covering with a cover slide. Samples were then incubated for 1 h at 37 °C in a humid box and washed once in PBS. Samples for the double staining of PTP and viability were mounted and analyzed as described in Section 4.8. 

### 4.7. Acrosomal Exocytosis

Slides were washed for 5 min twice in PBS and 50 µL of 15 µg/mL FITC-PNA and 0.0065 mg/mL Hoechst 33342 in PBS were added and covered with a 24 × 24 cover slide. The slides were then incubated for 30 min at room temperature in a humid box. Subsequently, the slides were washed in distilled water for 10 min and mounted with Fluoromount^TM^ aqueous mounting medium, sealed with nail-polish and examined in a fluorescence microscope Nikon Optiphot-2 (Nikon, Tokyo, Japan). At least 200 spermatozoa from two slides per sample were analyzed by blind counting using codified slides. Only completely reacted acrosomes were counted as reacted and spermatozoa showing partial staining in the acrosome were counted as unreacted.

### 4.8. Statistics

Statistical analysis was carried out using the computerized package GraphPad Prims 8.0.2 software for Windows (GraphPad Software, San Diego, CA, USA). The normality of the data was assayed with the Shapiro–Wilk test and the appropriate statistical assay was applied accordingly. Thus, repeated-measures ANOVA followed by Tukey post-hoc test or Kruskal–Wallis test followed by Dunn’s post-hoc test were employed for multiple comparisons, and paired one-tailed Student’s t-test or Wilcoxon matched-pairs signed-rank test were employed for paired comparisons. When one of the pairs was =0 we employed the one sample one-tailed sample pairing Student’s t-test or the Wilcoxon signed-rank test. The results were expressed as means ± SD. Significance was set at 0.05.

## Figures and Tables

**Table 1:** Kinetics of the spermatozoa during incubation under capacitating conditions before DGC, right after DGC (T0h) and every passing hour of incubation until T3 h. Abbreviations of CASA kinetics are detailed in the abbreviations section. Data are expressed as the mean ± SD.

Motility	Before DGC	T0h	T1h	T2h	T3h
VCL (µm/s)	76 ± 9	89 ± 11	88 ± 3	78 ± 10	71 ± 12
VAP (µm/s)	37 ± 4	41 ± 4	42.9 ± 1	40 ± 5	38 ± 8
VSL (µm/s)	27 ± 3	32 ± 3	37.7 ± 1	35 ± 4	34 ± 7
LIN (%)	37 ± 3 ^a^	38 ± 3 ^ab^	42 ± 1	46 ± 1 ^bc^	48 ± 2 ^c^
STR (%)	73 ± 7 ^a^	79 ± 3 ^ab^	85 ± 1 ^abc^	88 ± 1 ^bc^	89 ± 1 ^c^
WOB (%)	48 ± 3	46 ± 3	48 ± 2	51 ± 1	53 ± 3
BCF (Hz)	19 ± 0.7 ^a^	20.4 ± 0.4 ^ab^	22.4 ± 0.8	22.9 ± 0.8 ^bc^	24.1 ± 1 ^c^
ALH (µm)	2.9 ± 0.3	3.3 ± 0.3	3.38 ± 0.07	3 ± 0.07	2.8 ± 0.5
Motility (%)	56 ± 11 ^ab^	63 ± 9 ^a^	55 ± 11 ^ab^	46 ± 13 ^b^	43 ± 10 ^b^

Different letters indicate significant differences for each parameter among incubation times (*p* < 0.05, *n* = 6). DGC (density gradient centrifugation).

**Table 2:** Percentages of acrosomal exocytosis, plasma membrane integrity and protein tyrosine phosphorylation (PTP) before and after (time 0 of incubation) sperm processing by density gradient centrifugation (DGC) and after 3 h incubation under capacitating conditions. At times 0 and 3 h, samples were treated for 30 min with 10 µM of calcium ionophore A23187. Data are expressed as the mean ± SD.

Sample	Incubation (h)	Positive Acrosomal Exocytosis (%)	Intact Plasma Membrane (%)	PTP-Positive Sperm		
				Head (%)	Equatorial Region (%)	Whole Flagella (%)
Before DGC		4.2 ± 4.5 ^a^	58 ± 13 ^a^	40 ± 15 ^b^	31 ± 20 ^b^	1.4 ± 1.4 ^a^
Control	0	3.5 ± 1 ^a^	59 ± 6 ^a^	49 ± 18 ^ab^	38 ± 23 ^b^	1.4 ± 1.6 ^a^
Ionophore	0	4.8 ± 2.5 ^ac^	57 ± 10 ^a^	50 ± 15 ^ab^	43 ± 20 ^ab^	0.8 ± 1 ^a^
Control	3	9.3 ± 2.7 ^c^	27 ± 16 ^c^	61 ± 21 ^ac^	54 ± 28 ^ac^	9 ± 9.8 ^c^
Ionophore	3	12.6 ± 4.6 ^c^*	29 ± 13 ^c^	59 ± 17 ^ac^	52 ± 25 ^ac^	11 ± 12 ^c^

Different letters indicate significant differences at different incubation times under capacitating conditions (*p* < 0.05). An asterisk indicates significant differences between Control and ionophore (*p < 0.05, *n* = 6).

**Table 3:** Distribution of protein tyrosine phosphorylation in bull spermatozoa showing intact acrosome (IA) or acrosomal exocytosis (AE). Data are expressed as mean ± SD.

Incubation Time (h)	Ionophore Treatment	Acrosomal Exocytosis	Analyzed Spermatozoa (*n*)	PTP Patterns		
				I	II	III
Before DGC	no	IA	1356	64 ± 9 ^a^	24 ± 12 ^b^	12 ± 6 ^b^
		AE	60	78 ± 23 ^a^	12 ± 20 ^b^	12 ± 16 ^b^
0	no	IA	1269	63 ± 7 ^a^	29 ± 8 ^b^	8 ± 2 ^c^
		AE	44	65 ± 26 ^a^	30 ± 27 ^ab^	5 ± 9 ^b^
0	yes	IA	1410	56 ± 11 ^a^****	36 ± 14 ^b^***	8 ± 3 ^c^**
		AE	71	90 ± 14 ^a^	10 ± 14 ^b^	0
3	no	IA	1222	54 ± 11 ^a^*	36 ± 16 ^a^**	10 ± 7 ^b^*
		AE	130	82 ± 9 ^a^	15 ± 9 ^b^	3 ± 6 ^b^
3	yes	IA	1266	50 ± 13 ^a^**	39 ± 21 ^b^*	12 ± 8 ^c^**
		AE	184	83 ± 8 ^a^	11 ± 11 ^b^	6 ± 5 ^b^

Different letters indicate significant differences between the phosphorylation patterns within the same row (*p* < 0.05, *n* = 6). Asterisks indicate significant differences between reacted and no reacted of the same pattern (* *p* < 0.05, ** *p* < 0.01, *** *p* < 0.001, **** *p* < 0.0001, *n* = 6). DGC (density gradient centrifugation).

**Table 4:** Distribution of protein tyrosine phosphorylation in bull spermatozoa showing intact (I) or compromised (C) plasma membrane. Data are expressed as mean ± SD.

Incubation Time (h)	Ionophore Treatment	Plasma Membrane Integrity	Analyzed Spermatozoa (*n*)	PTP Patterns		
				I	II	III
Before DGC	no	I	711	59 ± 26 ^a^	40 ± 22 ^a^	0.1 ± 0.4 ^b^*
		C	510	51 ± 15 ^a^	47 ± 19 ^a^	1.4 ± 0.7 ^b^
0	no	I	688	46 ± 26 ^a^	43 ± 26 ^a^*	10 ± 22 ^b^
		C	474	30 ± 17 ^b^	63 ± 27 ^a^	7 ± 10 ^c^
0	yes	I	625	53 ± 20 ^a^**	44 ± 32 ^a^*	3 ± 3 ^b^
		C	451	28 ± 12 ^b^	68 ± 20 ^a^	4 ± 7 ^b^
3	no	I	318	35 ± 21 ^a^*	62 ± 20 ^a^*	2 ± 4 ^b^
		C	838	16 ± 6 ^b^	83 ± 13 ^a^	1 ± 1 ^c^
3	yes	I	339	51 ± 21 ^a^***	47 ± 21 ^a^***	2 ± 2 ^b^
		C	803	22 ± 16 ^b^	77 ± 16 ^a^	0.1 ± 0.4 ^c^

Different letters indicate significant differences between the phosphorylation patterns within the same row (*p* < 0.05, *n* = 6). Asterisks indicate significant differences between intact and compromised sperm plasma membrane within the same PTP pattern (* *p* < 0.05, ** *p* < 0.01 and *** *p* < 0.001, *n* = 6). DGC (density gradient centrifugation).

## Abbreviations

PTP	Protein tyrosine phosphorylation
I	Intact sperm plasma membrane
C	Compromised sperm plasma membrane
AE	Acrosomal exocytosis
IA	Intact acrosome
DGC	Density gradient centrifugation
PMI	Plasma membrane integrity
